# Advancing genome-based precision medicine: a review on machine learning applications for rare genetic disorders

**DOI:** 10.1093/bib/bbaf329

**Published:** 2025-07-16

**Authors:** Syed Raza Abbas, Zeeshan Abbas, Arifa Zahir, Seung Won Lee

**Affiliations:** Department of Precision Medicine, Sungkyunkwan University School of Medicine, Suwon 16419, Republic of Korea; Department of Precision Medicine, Sungkyunkwan University School of Medicine, Suwon 16419, Republic of Korea; Department of Artificial Intelligence, Sungkyunkwan University, Suwon 16419, Republic of Korea; Department of Biomedical and Robotics Engineering, Incheon National University, Songdo, Republic of Korea; Department of Precision Medicine, Sungkyunkwan University School of Medicine, Suwon 16419, Republic of Korea; Department of Artificial Intelligence, Sungkyunkwan University, Suwon 16419, Republic of Korea; Department of Metabiohealth, Sungkyunkwan University, Suwon 16419, Republic of Korea; Personalized Cancer Immunotherapy Research Center, Sungkyunkwan University School of Medicine, Suwon 16419, Republic of Korea; Department of Family Medicine, Kangbuk Samsung Hospital, Sungkyunkwan University School of Medicine, 29 Saemunan-ro, Jongno-gu Seoul 03181, Republic of Korea

**Keywords:** artificial intelligence, internet of things, machine learning, deep learning, healthcare, GBPM, explainable AI

## Abstract

Precision medicine tailors medical procedures to individual genetic overviews and offers transformative solutions for rare genetic conditions. Machine learning (ML) has enhanced genome-based precision medicine (GBPM) by enabling accurate diagnoses, customized treatments, and risk assessments. ML tools, including deep learning and ensemble methods, process high-dimensional genomic data and reveal discoveries in rare diseases. This review analyzes the ML applications in GBPM, emphasizing its role in disease classification, therapeutic optimization, and biomarker discovery. Key challenges, such as computational complexity, data scarcity, and ethical concerns, are discussed alongside advancements such as hybrid ML models and real-time genomic analysis. Security issues, including data breaches and ethical challenges, are addressed. This review identifies future directions, emphasizing the need for comprehensible ML models, increasing data-sharing frameworks, and global collaborations. By integrating the current research, this study provides a comprehensive perspective on the use of ML for rare genetic disorders, paving the way for transformative advancements in precision medicine.

## Introduction

### Background and motivation

Rare genetic disorders affect millions of people worldwide, presenting challenges in diagnosis and treatment. Fortunately, advances in genomic technology, particularly next-generation sequencing (NGS), have improved our ability to identify genetic causes, enhance diagnostic accuracy, and deepen our understanding of these conditions [1]. Machine learning (ML) and deep learning (DL) in particular has revolutionized genomic medicine by directly analyzing complex data to uncover patterns and relationships often missed by traditional approaches [2, 3].

In the clinical setting, ML enhances the diagnosis of rare genetic disorders by integrating genomic data with clinical phenotypes. ML models streamline diagnostics by prioritizing genes and predicting pathogenic variants, thereby reducing the time to diagnosis. Studies have highlighted the role of ML in improving the accuracy of variant interpretation in NGS-based diagnostics [4]. The benefits of ML extend beyond disease diagnosis to aiding in the creation of customized treatment options. By processing genomic and clinical data, ML models can identify potential treatment targets and predict patient responses to specific therapies [5].

Along with these advancements, the application of ML in genome-based precision medicine (GBPM) for rare genetic disorders faces several challenges. The scarcity of data is a major barrier because the rare nature of these conditions means that there are few high-quality annotated datasets available for training effective ML models. Moreover, the complexity of genomic data requires advanced computational methods to efficiently handle and analyze extensive information [6].

Recent advances have begun to address some of these challenges. The development of hybrid ML models that combine multiple data types, such as genomic, transcriptomic, and clinical data, has shown promise for improving predictive performance. Furthermore, real-time genomic analysis facilitated by ML algorithms is becoming increasingly feasible, enabling more timely and accurate clinical decision-making [7]. Table 1 presents the integration of ML and genomic technologies for investigating rare genetic disorders.

**Table 1:** Integration of ML and genomic technologies in rare genetic disorder research

**Category**	**Details**	**Impact**	**Examples**	**Challenges Addressed**	**Ref**
Genomic technologies (NGS)	Revolutionized variant identification, improving diagnostic accuracy.	Accelerates rare disorder diagnosis and understanding of genetic mechanisms.	Genome-wide association studies (GWAS).	Overcoming diagnostic delays.	[1]
ML in genomic medicine	Enhances diagnostic processes for rare genetic disorders; Identifies therapeutic targets.	Drives precision medicine by integrating and analyzing complex datasets.	Variant prioritization tools, gene prediction.	Data integration, improving diagnostic accuracy.	[8, 9]
Recent advancements	Hybrid ML models integrating multiple data types; Real-time genomic analysis.	Improves predictive accuracy, enables timely clinical decision-making, and broadens applications.	Multi-omics data integration, real-time prediction tools.	Enhances model performance and decision support in clinical settings.	[10]
ML in personalized medicine	Facilitates design of individualized therapeutic interventions.	Aligns with precision medicine to tailor medical care to patients’ unique characteristics.	Drug response prediction, target identification.	Addresses variability in patient responses to treatment.	[11]

#### Overview of ML in GBPM

The integration of ML into GBPM has significantly advanced the diagnosis and treatment of rare genetic disorders. ML algorithms, particularly DL models, excel at analyzing complex genomic data, identifying patterns that facilitate accurate disease classification, and discovering new biomarkers [12, 13]. In clinical diagnostics, ML improves the interpretation of genomic variants by predicting their pathogenicity, thereby facilitating g the identification of generative mutations in patients with rare diseases. For example, AI-MARRVEL, an ML system developed to prioritize potentially causal variants for Mendelian disorders, has shown improved diagnostic efficiency [14, 15].

**Figure 1:**
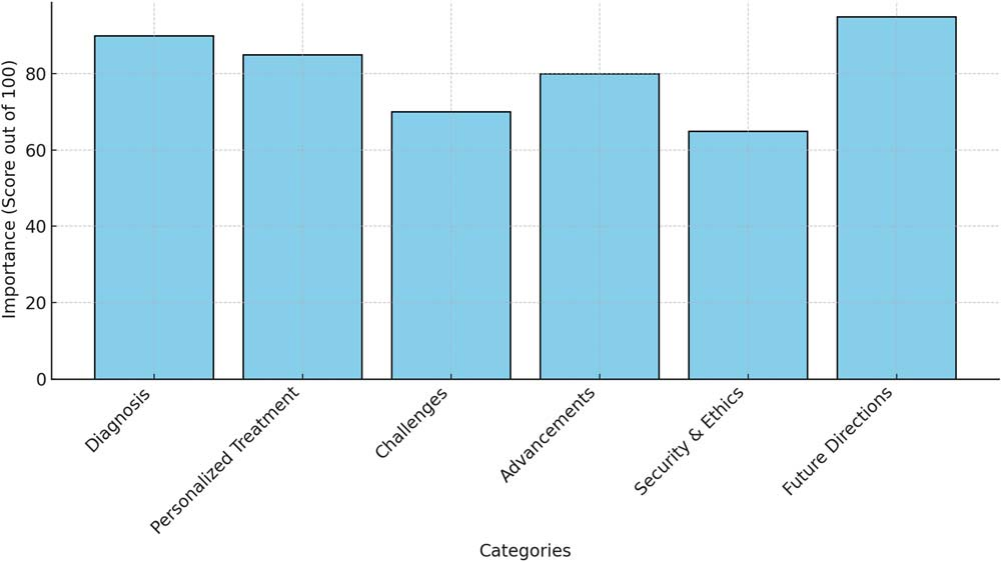
Relative importance of key thematic areas in ML applications to GBPM (2023–2024).

Security concerns, including the risk of data breaches and ethical crises in genomic data sharing, are also major problems. Therefore, it is important to use strong encryption methods and develop thorough policy frameworks to minimize such risks [16]. The development of explainable ML models, enhanced data-sharing frameworks, and global collaboration is vital for advancing GBPM. These efforts aim to overcome existing barriers and fully realize the capability of ML to improve the diagnosis and treatment of rare genetic disorders [17]. Figure 1 presents the importance scores of the key aspects of ML in GBPM. The updated references from 2023 align with the key aspects of ML in GBPM [18–21].

### ML in medicine

ML has become a transformative force in medicine, enhancing diagnostic accuracy, personalizing treatments, and streamlining healthcare operations.

#### Diagnostic accuracy and disease prediction

ML algorithms have transformed disease diagnosis by enabling early and accurate detection. In 2023 and 2024, ML models have demonstrated an exceptional ability to identify complex patterns in medical imaging data, particularly for diseases such as diabetes, cancer, Alzheimer’s disease, and cardiovascular disorders [22–24].

#### Personalized treatment plans

Procedures in customized medicine are altered according to the unique genetic, environmental, and lifestyle characteristics of patients. ML has advanced this approach by analyzing diverse datasets to predict specific patient responses to therapies [25]. In 2024, ML-enabled systems were increasingly used to design individualized care pathways. These tools consider possibilities, drug interactions, and patient preferences to provide clinicians with actionable awareness.

#### Drug discovery and development

ML enhances the modification of current drugs by identifying new applications based on shared molecular processes. These inventions underscore the potential of ML to address the lack of medical needs while optimizing resource allocation in the drug industry. Additionally, recent studies have employed ML models to predict off-target effects and therapeutic efficacy, leading to the discovery of new uses for approved drugs [26].

The integration of ML with drug discovery has enhanced the identification and development of new therapies. Algorithms can rapidly screen billions of molecular structures to identify potential drug candidates, thereby significantly reducing the time and costs associated with traditional drug development methods [27].

#### Operational efficiency in healthcare

ML enhances clinical workflows. Decision aid systems use real-time data to prioritize urgent cases, ensuring timely care.ML enhances clinical workflows. Decision-aid systems use real-time data to prioritize urgent cases and ensure timely care. For instance, AI-driven triage tools can analyze symptoms and vital signs to predict patient outcomes, enabling healthcare providers to allocate resources more effectively. These applications demonstrate how ML contributes to a more efficient and patient centric healthcare system [28, 29].

ML has improved several administrative tasks in healthcare, improving operational efficiency. Automated scheduling systems powered by ML optimize patient appointments, minimize waiting times, and enhance resource utilization [30].

#### Ethical considerations

The adoption of ML in medicine faces significant challenges. Data privacy remains a critical issue because ML models require access to vast amounts of sensitive information. Ensuring that patient data are secure and ethically used is important, especially with the increasing number of reports of data breaches. Furthermore, algorithmic bias can lead to disparities in healthcare outcomes, underscoring the need for fair and transparent model development [31–33]. Table 2 summarizes recent advancements and challenges of ML in medicine. It details specific areas, such as diagnostic accuracy, personalized treatment plans, and drug discovery, highlighting developments from 2023 to 2024 and providing references for each.

**Table 2:** Recent advancements and challenges of ML in medicine

**Area**	**Description**	**Recent developments (2023–2024)**	**Ref**
**Diagnostic accuracy and disease prediction**	ML models are transforming disease diagnosis by recognizing complex patterns in clinical and imaging data. These models have proven particularly effective in diagnosing diseases such as cancer, diabetes, and cardiovascular disorders.	DL models for predicting RNA-Seq expression in tumors for cancer diagnostics.	[34, 35]
**Personalized treatment plans**	ML has enhanced personalized medicine by predicting patient-specific responses to therapies. This approach tailors interventions to genetic, environmental, and lifestyle factors, improving treatment outcomes.	Genomic data-driven treatment predictions in oncology. Systems that design individualized care pathways, including drug interactions and patient preferences.	[36, 37]
**Drug discovery and development**	ML is accelerating drug discovery by screening molecular structures and optimizing drug development, reducing time and costs. It is also advancing drug repurposing by predicting new uses for existing medications.	AI-driven drug design for diseases like Parkinson’s and autoimmune disorders. ML for predicting off-target effects and therapeutic efficacy.	[38, 39]
**Operational efficiency in healthcare**	ML improves healthcare operations by automating administrative tasks and streamlining clinical workflows. It enhances scheduling, resource allocation, and care delivery.	Automated patient scheduling to reduce wait times. Real-time decision-support systems prioritizing urgent cases.	[40, 41]
**Challenges and ethical considerations**	Data privacy, algorithmic bias, and ethical concerns such as informed consent and accountability are challenges in adopting ML in healthcare.	Ongoing research to mitigate data privacy risks. Efforts to address algorithmic bias and ensure model transparency.	[42, 43]

### Opportunities

ML algorithms have the potential to improve the diagnosis of rare genetic disorders by identifying subtle patterns in genomic data that might be overlooked by traditional methods. For instance, AI-MARRVEL was developed to prioritize variants that could cause Mendelian disorders, thus streamlining the diagnostic process [9].

**Figure 2:**
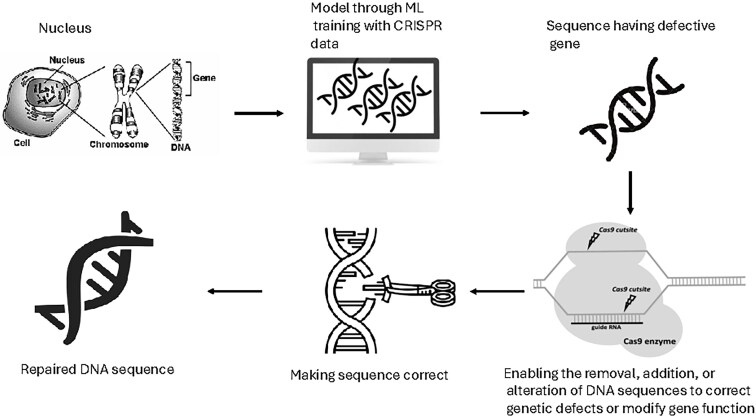
A theoretical representation of CRISPR gene editing utilizing ML computational model.

The combination of AI and CRISPR technologies has the potential to revolutionize drug discovery by enabling the rapid identification of therapeutic targets and the development of new treatments for genetic disorders. This collaboration could lead to more effective and efficient therapeutic interventions [44]. Figure 2 illustrates CRISPR, which is the most versatile, affordable, and user-friendly genome-editing technology available today. ML and DL algorithms are integrated to enhance its efficiency and precision [45, 46].

### Scope and purpose of this review

The scope and purpose of this review article is to consolidate and advance our understanding of the intersection of ML, genomics, and precision medicine, particularly in addressing rare genetic disorders. Rare genetic disorders represent a significant challenge in healthcare, and are characterized by the complexity of diagnosis, limited treatment options, and small number of affected individuals. This review aims to provide a comprehensive perspective on how ML technologies can enhance GBPM and improve outcomes under these conditions. Table 3 reveals the distinctiveness of this review by comparing the details of its scope, advanced methodologies, comprehensively addressed challenges, identified knowledge gaps, and unique contributions with other articles in the field.

**Table 3:** Comparison of this review with existing review articles on ML in GBPM for rare genetic disorders

**Ref**	**Scope**	**Methodological advances**	**Challenges/limitations**	**Knowledge gaps**	**Unique contributions**
[47]	General ML in healthcare	Standard ML techniques	Data scarcity and complexity	Overlook ethical issues	General applications in healthcare without focus on rare disorders
[48]	Genomics broadly	Basic genomics applications	Computational demands, general ethical concerns	Lack of real-world applications	Broad discussion, no specific focus on rare disorders
[49]	Precision medicine	Some new methodologies like AI	Ethical concerns, data integration challenges	Standardization issues	Advances in precision medicine without specific ML focus, Specially work on cancer
[50]	Broad ML applications in various diseases	Traditional diagnostic tools	General scalability issues, lack of interpretability	Overlooks rare genetic disorders	Diverse but not specific to rare genetic disorders
[51]	ML and data science in medicine	Basic ML applications	Data heterogeneity, lacks depth in clinical integration	Misses interdisciplinary insights	Focuses on data science without integrating clinical insights
**This review**	ML in GBPM specifically for rare genetic disorders	Advanced ML methodologies like XAI, real-time genomic tools	Addressing data scarcity, computational complexity, ethical concerns comprehensively	Identifies critical gaps in rare disorders	Interdisciplinary insights, advanced methodologies, specific focus on clinical implementation and policy implications

#### Scope of the review

This review covering multiple disciplines, including clinical medicine, computational biology, genomics. It explores how advancements in ML have been adapted to solve problems unique to rare genetic disorders such as data scarcity and genomic complexity.

The review investigates specific applications of ML, including:

- Enhanced diagnostic processes for identifying pathogenic genetic variants;
- The development of personalized treatment regimens based on individual genomic profiles;
- Accelerated drug discovery and repurposing using ML to identify potential therapeutic targets.

A significant part of the scope includes an evaluation of the barriers to implementing ML in GBPM. These barriers include data heterogeneity, computational demands, ethical issues, and the need for interpretability in clinical settings. This review highlights the recent advancements in ML methodologies, such as explainable AI (XAI), hybrid ML models integrating multi-omics data, and real-time genomic analysis tools that enhance clinical decision-making.

#### Purpose of the review

By synthesizing research findings from 2020 to 2025, this review aims to provide an updated and cohesive understanding of the current state of ML in GBPM. It seeks to bridge gaps in the literature by addressing underexplored areas such as real-world implementation challenges and ethical considerations.

This review seeks to identify the knowledge and practice gaps that hinder the full potential of ML in this field. This review serves as a roadmap for future research by proposing the following actionable recommendations:

- Develop interpretable and clinically applicable ML models;
- Enhance data-sharing frameworks to facilitate global collaborations; and
- Explore novel algorithms to handle high-dimensional genomic data efficiently.

This review underscores the importance of aligning advancements in ML with ethical guidelines and regulatory frameworks to ensure equitable and secure use of genomic data. By addressing these aspects, this review aims to inform healthcare policymakers and regulatory bodies.

### Contributions to the literature

This section explains how this study extends the existing knowledge, addresses gaps, and provides novel insights into the application of ML in GBPM for rare genetic disorders.

One of the primary contributions of this study is its ability to bridge gaps in the existing literature. Despite significant advances in ML and genomics, the application of these technologies to rare genetic disorders remains limited. This study merges scattered findings and presents a cohesive viewpoint, offering a comprehensive overview that was previously unavailable. A review of diverse case studies provides insights into specific applications, such as biomarker discovery, disease classification, and personalized treatments. This study reveals the importance of interdisciplinary methods by integrating insights from each field, which demonstrates how ML can act as a bridge between computational data analysis and clinical applications, encouraging collaborative efforts among researchers from diverse backgrounds.

Another main contribution of this study is the identification and evaluation of advanced ML techniques, including DL, hybrid models, and ensemble approaches, for rare genetic disorders. This paper not only discusses their theoretical applications but also provides practical insights into their implementation challenges, clinical relevance, and performance metrics. This paper systematically discusses the challenges associated with ML in GBPM, including data scarcity, computational complexity, and ethical problems. In contrast to many studies focusing solely on technological aspects, this review integrates discussions on regulatory and ethical implications, offering a holistic view of the field. Moreover, it proposes actionable recommendations such as enhanced data-sharing frameworks, XAI models, and standardized protocols for genomic data management. In addition to its academic contributions, this study has practical importance for healthcare professionals, technology developers, and policymakers. The demonstration of how ML can improve diagnostic accuracy, accelerate drug discovery, and personalize treatment illustrates the transformative potential of these technologies in clinical settings. This practical focus ensures that the research is not only theoretically robust, but also actionable.

## Methodology

**Figure 3:**
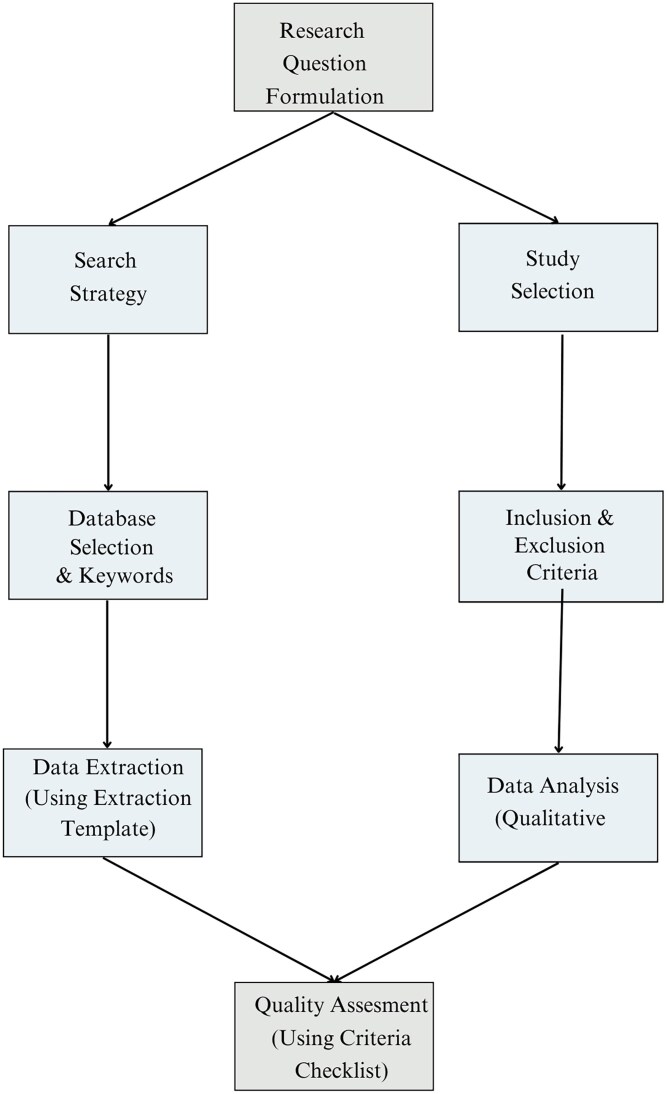
Workflow diagram for the systematic review methodology.

This section defines the structured and strict approach employed in this review to analyze the intersection of ML and GBPM for rare genetic disorders. An extensive workflow for the systematic review, which follows PRISMA guidelines, is shown in Fig. 3, which includes every stage from database identification to the ultimate decision to include specific research studies.

### Research questions

This review addresses the critical aspects of the application of ML in GBPM by formulating the following research questions:

- What are the current applications of ML in GBPM for rare genetic disorders?
- What are the challenges and opportunities related to integrating ML in this domain?
- What tools, advancements, and methodologies have emerged in recent years and what are the future research directions?

These questions were designed to provide a comprehensive understanding of the field and to guide the selection and analysis of relevant studies.

### Search strategy

A multistage search strategy was adopted to ensure the inclusion of relevant high-quality literature.

- Database Selection: The review utilized academic databases, including PubMed, Scopus, IEEE Xplore, MDPI, and Google Scholar. These platforms were selected for their extensive inclusion of biomedical, computational, and engineering studies.
- Keyword Formulation: Combinations of the following list of terms was used to maximize the retrieval of the relevant studies.
  - “ML in Precision Medicine”
  - “Genomics and ML”
  - “Rare Genetic Disorders and AI”
  - “Genome-Based Diagnostics”
- Timeframe: To capture recent advancements while acknowledging foundational studies, the search was restricted to articles published between 2020 and 2025.
- Additional Sources: To ensure comprehensiveness, manual searches were conducted on the references of the selected articles and key journals.

**Figure 4:**
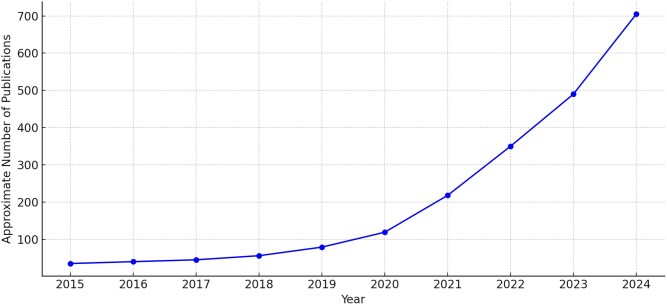
Annual publication trends (2015–2024) in ML applications for GBPM in rare genetic disorders.

Figure 4 presents the approximate number of peer-reviewed papers (journal articles, conference papers) published annually between 2015 and 2024 on ML applications in genome-driven precision medicine for rare genetic disorders. This figure was compiled from bibliometric analyses of PubMed-indexed literature and cross-disciplinary databases (e.g. Web of Science, IEEE Xplore, Google Scholar).

### Study selection

The selection process involved two phases.

**Phase 1**—Title and Abstract Screening: Articles were screened for relevance based on their titles and abstracts. Studies addressing ML in GBPM and rare genetic disorders were added to the list of candidates.

**Phase 2**—Full Text Review: Candidate articles were reviewed in full to assess their alignment with the research objectives and inclusion criteria. The inclusion and exclusion criteria ensured that only relevant high-quality studies were included.

Table 4 shows the summary of the studies retrieved, screened, excluded, and finally included.

**Table 4:** Summary of study selection process following PRISMA guidelines

**Stage**	**Number of articles**
Records identified through database searching	865
– PubMed	220
– Scopus	185
– IEEE Xplore	145
– Google Scholar	315
Additional records identified through manual search	42
Total records before duplicates removed	907
Duplicates	132
Records after duplicates removal	775
Records excluded after title/abstract screening	534
Full-text articles assessed for eligibility	241
Full-text articles after applying exclusion criteria	172
Studies included in final systematic review	69

**Inclusion Criteria**

- The study focused on ML applications in genomics and rare genetic disorders.
- The study was a peer-reviewed journal article or conference proceeding.
- The publication had sufficient methodological details and reproducible results

**Exclusion Criteria**

- The study was a non-peer-reviewed articles, opinion pieces, or grey literature
- The study had incomplete or ambiguous methodologies.
- The article was unrelated to ML, genomics, or rare genetic disorders.

### Data extraction

A standardized data extraction protocol was developed to ensure consistency and comprehensiveness. The key details extracted from each study are as follows:

- Research objectives and hypotheses.
- Types and sources of genomic data utilized in the study.
- ML methodologies and models (e.g. DL, ensemble methods).
- Evaluation metrics used to assess model performance.
- Key findings, limitations, and future directions proposed by the authors.

### Quality assessment

The quality of each study was rigorously evaluated using a modified Critical Appraisal Skills Programme checklist. The key assessment criteria were as follows:

- Clarity and specificity of the research objectives
- Robustness and reproducibility of the methodologies
- Appropriateness of the evaluation metrics and datasets
- Consideration of the ethical and regulatory issues in ML applications

Only studies meeting high-quality standards were included in the final synthesis.

## Overview of ML in GBPM

ML has emerged as a revolutionary technology in GBPM, offering novel solutions for the treatment, diagnosis, and understanding of rare genetic disorders. This section provides an extensive overview of the key applications, challenges, advancements, case studies, and future opportunities of ML in this transformative field.

### Key applications of ML in GBPM

ML has enabled significant advancements in several critical areas of GBPM, and algorithms play a pivotal role in analyzing high-dimensional genomic data to identify pathogenic variants linked to rare genetic disorders. CNNs and RNNs have been used to detect structural variations, single nucleotide polymorphisms, and copy number variations [52].

Biomarker discovery is crucial for understanding disease progression and designing targeted therapies [53].

The application of ML to drug discovery has significantly reduced the time and cost associated with the development of therapies for rare diseases [54].Moreover, ML-driven drug repurposing frameworks have successfully suggested new therapeutic uses for existing drugs such as antiepileptic medications for treating rare mitochondrial disorders [55].

### Advancements in ML for genomics

Advancements in computational productivity have enabled real-time analysis of genomic data. High-performance ML algorithms are used in clinical settings to rapidly identify actionable genetic mutations and ensure timely intervention for critical conditions, such as neonatal genetic disorders [56]. Table 5 provides an overview of the diverse applications of ML in GBPM, detailing the techniques used and specific examples of their implementation.

**Table 5:** Applications of ML in GBPM

**Application**	**Techniques**	**Examples**	**References**
Disease diagnostics	CNNs, RNNs	Duchenne Muscular Dystrophy, Fragile X Syndrome	[57, 58]
Biomarker discovery	Random Forest, Feature Selection	Rare cancers, metabolic disorders	[59, 60]
Therapeutic target identification	GNNs, GANs	ALS, spinocerebellar ataxia	[61, 62]
Drug repurposing and development	Autoencoders	Anti-epileptic drugs for mitochondrial disorders	[63, 64]
Personalized treatment optimization	Reinforcement learning	Cystic fibrosis	[65, 66]

### Case studies in ML applications

Several case studies illustrate the transformative impact of ML in GBPM:

1. ML in Rare Disease Diagnosis: A CNN-based approach was successfully employed to identify pathogenic variants of Rett Syndrome.
2. Drug Repurposing for Rare Disorders: A DL model identified novel therapeutic uses of the anti-inflammatory drug celecoxib in treating rare mitochondrial disorders, demonstrating a cost-effective approach to drug discovery [67].
3. Multi-Omics Integration in Rare Cancer Biomarker Discovery: A hybrid ML model integrating genomic, transcriptomic, and proteomic data identified biomarkers for early detection of rare sarcomas, enhancing survival rates through timely intervention [68, 69].

### Opportunities for future research

Creating standardized and accessible genomic datasets across diverse populations will address data scarcity and improve model performance [70]. By integrating ML with cutting-edge technologies such as CRISPR and single-cell sequencing, researchers can pave the way for groundbreaking discoveries in therapeutic development [71]. Furthermore, the growing availability of patient-specific genomic data will enable highly personalized treatment approaches, further advancing precision medicine [72]. In this context, Table 6 highlights the key advancements in ML for genomics, detailing the innovative techniques employed and their transformative impact on genomic research.

**Table 6:** Advancements in ML for genomics

**Advancement**	**Techniques**	**Impact**
Integration of multi-omics Data	VAEs	Predicting rare disease phenotypes
XAI in genomics	XAI frameworks	Interpretable insights into gene mutations
Real-time genomic analysis	High-performance ML algorithms	Rapid identification of genetic mutations

## Security issues

This section discusses the complexities of genomic data privacy, cybersecurity threats, algorithmic security, and governance. In addition, it presents actionable strategies for mitigating these challenges.

### Privacy concerns in genomic data

Genomic data are highly sensitive, as they uniquely identify individuals and reveal information about hereditary conditions, predispositions to diseases, and even ancestral origins. The impact of these data extends to family members and future generations, making breaches particularly harmful. Unauthorized access could lead to genetic discrimination, in which employers, insurers, or third parties exploit this information to deny opportunities or coverage [73]. ML algorithms can combine anonymized genomic data with external datasets (e.g. demographic data, clinical records) to reverse engineer identifiable information. This reduces the effectiveness of traditional anonymization approaches [74].

### Cybersecurity threats

Genomic databases are prime targets for cyberattacks because of their immense value. A breach exposes not only personal data, but also valuable research insights that could be exploited for financial gain or industrial espionage. For example, stolen genomic data can be sold on the black market for purposes ranging from identity theft to biological weapon development [75]. Ransomware attacks are increasingly targeting healthcare and research institutions. These attacks involve encrypting the genomic data and demanding payment for decryption. Such incidents disrupt critical research and delay clinical applications, jeopardizing patient care and institutional reputations [76].

Table 7 summarizes the security issues, their impacts, mitigation strategies, and key studies in ML for genomic precision medicine.

**Table 7:** Security issues in ML for genomic precision medicine

**Aspect**	**Challenge**	**Impact**	**Mitigation Strategy**	**Ref**
Genomic data sensitivity	Unauthorized access & discrimination	Harm to individuals and families	Enhanced data anonymization and encryption methods	[77]
Re-identification risks	Advances in de-anonymization techniques	Compromise of privacy protections	Implementation of stricter access controls and continuous privacy assessments	[78]
Cross-border data exchange	Differing international data protection laws	Legal and ethical compliance issues	Development of international data sharing agreements	[79]
Cybersecurity vulnerabilities	High-value targets for cyberattacks	Financial and data losses	Application of state-of-the-art cybersecurity technologies	[80]
Ethical and regulatory compliance	Complexity of ethical considerations and laws (e.g. GDPR, HIPAA)	Barriers in international research cooperation	Advocacy for and implementation of global ethical standards	[81]
Algorithmic biases	Bias in data sets leads to skewed ML outcomes	Inequitable healthcare outcomes	Use of diverse datasets and bias mitigation algorithms	[82]
Adversarial attacks	Manipulation of ML models via data inputs	Erroneous model outputs affecting healthcare decisions	Deployment of adversarial robustness techniques and model validation processes	[83]

### Ethical and legal

Regulations, such as the General Data Protection Regulation (GDPR) in Europe and the Health Insurance Portability and Accountability Act (HIPAA) in the United States, impose strict requirements on data security and privacy. Although these frameworks aim to protect individuals, their regional nature poses challenges for global genomic collaboration. For example, genomic data collected in one jurisdiction may not meet the compliance standards of another, thus impeding cross border research [84].

Obtaining meaningful informed consent has become increasingly complex in genomic research, particularly when ML models are used. Participants may not fully understand how their data will be analyzed, shared, or used in secondary applications. Long-term storage of genomic data for future research adds another layer of complexity, as participant consent may not extend indefinitely [85]. Genomic datasets often underrepresent minority populations, leading to biases in ML models. This lack of diversity results in algorithms that perform poorly for underrepresented groups, thereby exacerbating healthcare disparities [86].

### Algorithmic security

Adversarial attacks, in which input data are subtly modified to deceive ML models, represent a growing threat. For instance, adversarial perturbations in genomic sequences fed to an ML model can result in the misclassification of genetic variants. This is particularly concerning in clinical settings, where ML predictions inform critical medical decisions [87].

**Figure 5:**
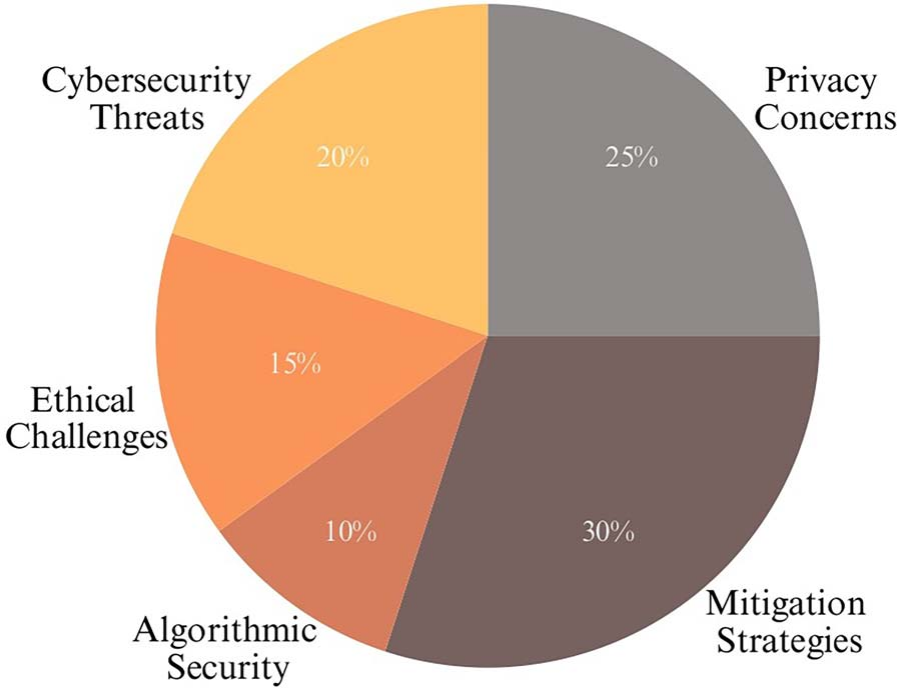
Proportional focus on genomic data security challenges.

Figure 5 visually represents the focus on various genomic data security challenges, emphasizing that categories such as mitigation strategies and privacy concerns are key priorities in addressing security issues [88–90].

### Mitigation strategies

Cutting-edge encryption methods, such as homomorphic encryption and secure multiparty computation, allow encrypted data to be computed without revealing the raw data. These methods enable collaborative research while maintaining stringent data privacy [91].

FL is a decentralized procedure that trains ML models across multiple institutions without transferring raw genomic data [88]. Blockchain technology provides immutable audit trials for genomic data. Each transaction involving data access or modification is transparently recorded, ensuring accountability and preventing unauthorized changes [92].

**Figure 6:**
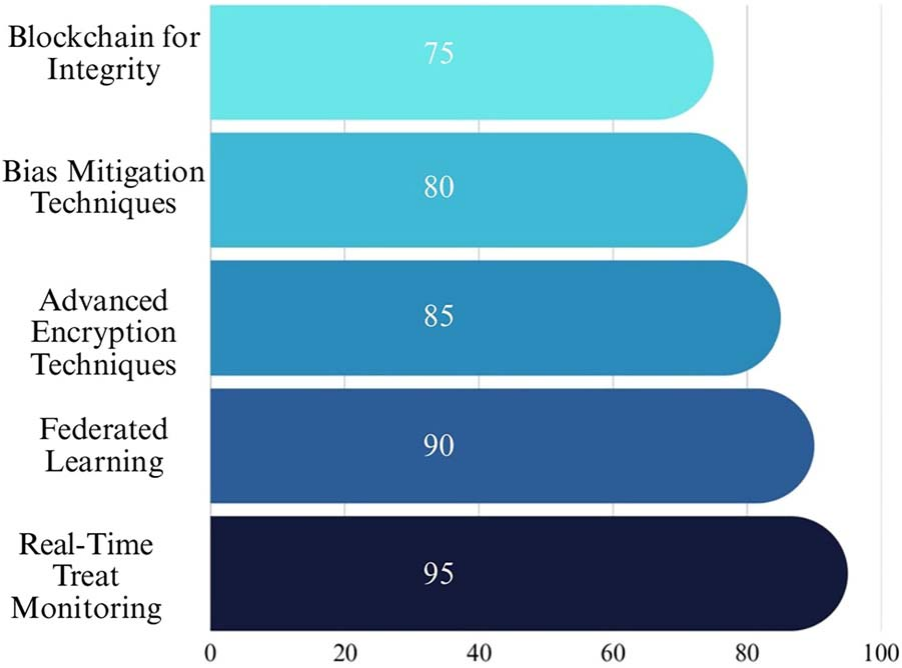
Importance of mitigation strategies in genomic ML security.

Figure 6 shows the relative importance of various mitigation strategies in genomic ML security, emphasizing their roles in enhancing data protection and integrity. These strategies collectively address the pressing challenges in genomic data security, aligning with emerging global standards [88, 89, 93–95].

## Advancements in GBPM

This section explores the key advancements that have transformed GBPM into the cornerstone of modern healthcare.

### NGS technologies

The advent of NGS technology has been revolutionary for GBPM. NGS platforms now enable the rapid sequencing of entire genomes with high accuracy, significantly reducing costs and timeframes. For instance, third-generation sequencing methods such as single-molecule real-time (SMRT) sequencing and nanopore sequencing provide long-read data that resolve structural variations and complex genomic regions more effectively than the earlier short-read technologies [96].

NGS has paved the way for routine clinical applications such as the diagnosis of rare genetic disorders, identification of cancer mutations, and guidance of personalized therapies. Whole-genome sequencing (WGS) and whole-exome sequencing are increasingly integrated into diagnostic workflows, enabling the detection of pathogenic variants with unprecedented precision [97].

**Figure 7:**
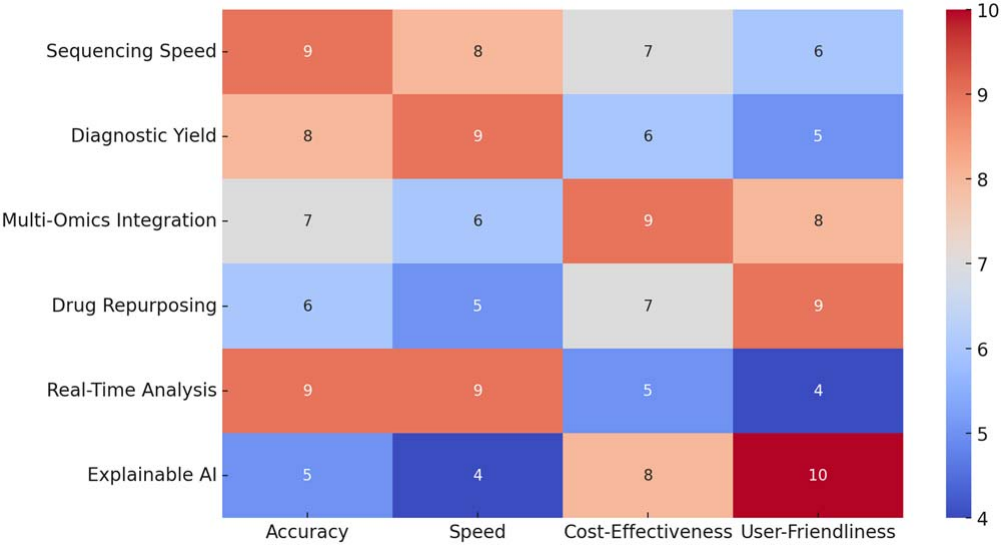
Impacts of technologies on different aspects in precision medicine.

Figure 7 shows the varying impacts of different technological advancements in GBPM on crucial aspects such as accuracy, speed, cost effectiveness, and user friendliness.

### Integration of multi-omics data

The integration of genomic, transcriptomic, proteomic, and metabolomic data has opened new avenues for understanding complex diseases. Multi- omics approaches provide a holistic view of biological systems, revealing interactions between genes, proteins, and metabolites that drive disease mechanisms [98].

ML algorithms play a pivotal role in integrating and analyzing multi- omics datasets. Advanced techniques such as GNNs and autoencoders are now used to identify key biomarkers and regulatory pathways [60].

### ML in precision diagnostics

ML models have transformed the identification and clarification of pathogenic variants in genomic data. Tools such as DeepVariant, PolyPhen, and MutPred use DL algorithms to classify genetic mutations as benign or pathogenic with high precision. These advancements are particularly beneficial for diagnosing rare genetic conditions for which conventional methods may fall short [99].

ML-powered precision diagnostics have significantly improved the diagnostic yield for rare diseases. For example, ensemble learning methods that combine multiple algorithms outperform traditional single-method approaches in identifying disease-causing mutations [100].

### Advancements in therapeutics and drug development

Recent advancements in genome editing methodologies such as CRISPR-Cas9 and base editing have revolutionized therapeutic development. ML models have been used to predict off-target effects, optimize guide RNA design, and identify potential therapeutic targets [101].

ML has accelerated drug discovery and repurposing by enabling the identification of novel therapeutic uses for existing drugs. Algorithms trained on genomic and chemical databases have been proposed as potential treatments for rare metabolic and neurological disorders. These advancements have reduced the time and costs associated with traditional drug development processes [102].

### XAI in genomics

The rise of the XAI has addressed the black-box nature of traditional ML models. XAI methods provide interpretable insights into the link between specific genetic variants and disease phenotypes. This has improved clinician trust and facilitated regulatory approval of AI-driven diagnostic tools [103]. XAI models have enhanced the discovery of biomarkers by highlighting the most relevant features of multi-omics data. For instance, SHAP (SHapley Additive exPlanations) values are used to rank the importance of genetic and epigenetic markers in predicting disease outcomes, guiding targeted therapeutic development [104].

The integration of XAI into genomic applications is essential to address the black-box nature of traditional ML models. Future research should focus on designing interpretable models that provide actionable insights, such as highlighting the most relevant genetic features contributing to disease prediction [105, 106]. [106].

Techniques such as SHAP, LIME (Local Interpretable Model-Agnostic Explanations), and integrated gradients are increasingly used to provide insight into feature importance, allowing researchers to understand which genes, SNPs, or regulatory elements influence predictions. These methods are essential in clinical genomics, where explainability fosters trust and facilitates biological discovery. However, most XAI tools were originally developed for generic tabular or image data and may not capture the hierarchical and interdependent nature of genomic information. Genomic data often involves intricate biological networks, nonlinear gene interactions, and complex regulatory effects that challenge the assumptions of many XAI methods [107, 108].

Future research should focus on developing fairness-aware algorithms that can detect and mitigate biases in training datasets. Techniques such as adversarial debiasing, resampling, and reweighting ensure that ML predictions are equitable across diverse demographic groups. Multi-omics integration is critical for understanding complex disease mechanisms. Future studies should focus on hybrid ML models that combine genomic, transcriptomic, proteomic, and epigenomic data to provide a holistic view of biological systems [98].

Table 8 provides a detailed overview of the significant advancements in GBPM, categorizing them by technology, applications, benefits, and examples across various subcategories.

**Table 8:** Detailed overview of advancements in GBPM

**Category**	**Subcategory**	**Technology/Method**	**Applications**	**Benefits**	**Examples/Details**
NGS technologies	Enhanced sequencing	SMRT, nanopore	Whole genome sequencing	High accuracy, speed	Resolves structural variations
	Clinical applications	NGS platforms	Diagnostics, personalized therapies	Increased diagnostic yield	Cardiomyopathies, Mitochondrial Disorders
Multi-omics data integration	Comprehensive insights	Multi-omics (Genomic, etc.)	Disease mechanism analysis	Holistic view of biological systems	Reveals gene-protein-metabolite interactions
	ML integration	GNNs, autoencoders	Biomarker identification	Improves accuracy of disease classification	Predicts disease phenotypes
ML in diagnostics	Variant prediction	DeepVariant, PolyPhen	Mutation classification	High precision	Classifies genetic mutations
	Diagnostic yield improvement	Ensemble learning	Rare disease diagnosis	Better performance in complex cases	Identifies disease-causing mutations
Therapeutics and drug development	Target identification	CRISPR-Cas9, base editing	Gene editing	Precise, safe	Optimizes guide RNA design
	Drug repurposing	ML	Drug discovery	Reduces development time and cost	Suggests new therapeutic uses for existing drugs
Real-time genomic analysis	High-performance computing	Distributed computing	Large dataset analysis	Enables real-time processing	Analyzes terabytes of data within hours
	Clinical decision-making	Rapid WGS	Neonatal intensive care diagnostics	Timely interventions	Diagnoses genetic disorders in critically ill infants
XAI in genomics	Trust and adoption	XAI	AI model interpretability	Improves clinician trust	Explains genetic variant-disease links
	Biomarker discovery	SHAP values	Biomarker identification	Guides targeted therapeutic development	Ranks importance of genetic markers
Emerging technologies	Genome editing	CRISPR, ML	Treating genetic disorders	Precise editing protocols	Used in sickle cell and Duchenne muscular dystrophy
	Single-cell genomics	ML	Cellular heterogeneity analysis	Identifies rare cell populations	Personalized therapies in cancer and immunology

### Large language models for genomics

Large Language Models (LLMs), which have demonstrated exceptional performance in natural language processing, remain underexplored in the domain of genomic sequence analysis. Unlike traditional ML approaches that rely heavily on handcrafted features and domain-specific encodings, LLMs offer a unique capacity to model complex dependencies and long-range interactions in biological sequences. For instance, models like DNABERT treat DNA sequences as a language, applying bidirectional transformers to k-mer tokenized input and learning context-aware representations for genomic tasks such as promoter identification and splice site detection [109]. Despite their promise, the integration of LLMs in genomics has been limited, often overshadowed by conventional convolutional or recurrent models. This gap presents a fertile area for research, particularly as sequence databases grow exponentially and the need for interpretable, high-capacity models intensifies.

Emerging models like the Nucleotide Transformer, which scales to billions of parameters and has been pretrained on massive nucleotide datasets, demonstrate the feasibility of adapting large-scale transformer architectures for biological applications [110]. These models not only capture biologically relevant patterns without supervision but also exhibit strong transferability across diverse tasks such as variant effect prediction, chromatin accessibility modeling, and evolutionary conservation estimation. Introducing a dedicated subsection on LLMs for genomic analysis would significantly enrich the discourse on advanced computational approaches in bioinformatics. As the field transitions toward more data-intensive and hypothesis free frameworks, LLMs offer a compelling paradigm shift moving from manually curated pipelines to end-to-end learnable systems capable of extracting nuanced biological insight.

## Limitations

Despite significant advancements in GBPM, several limitations have hindered its full realization. These obstacles span the technical, ethical, regulatory, and practical domains, thereby affecting the adoption and effectiveness of ML and genomic technologies. This section explores these limitations in detail, highlighting the complexities and limitations of implementing precision implementation.

### Data-related limitations

One of the primary challenges of GBPM is the limited availability of high-quality and diverse genomic datasets. Rare genetic disorders inherently suffer from small sample sizes, which restricts the ability of ML models to generalize effectively. This lack of diversity not only exacerbates healthcare disparities but also reduces the global applicability of ML-driven insights. Addressing data imbalance issues requires a concerted effort to curate diverse and representative dataset, alongside techniques such as data augmentation and synthetic data generation to balance the training data [86, 111].

The quality of genomic data can vary significantly, with issues such as sequencing errors, missing values, and noise affecting the model reliability. Variability in sequencing platforms, data pre-processing pipelines, and annotation standards introduces heterogeneity, which complicates data integration and analysis [112].

### Algorithmic and computational limitations

Analysis of high-dimensional genomic data requires substantial computational resources, including high-performance computing systems and optimized algorithms. This demand increases further when integrating multi-omics data or scaling analyses to population-level datasets. Institutions with a limited computational infrastructure, particularly in low- and middle-income countries, face significant barriers to participating in genomic research [113].

Many ML models, particularly DL architectures, have been criticized for their lack of interpretability. These black-box models provide predictions without explaining the underlying reasoning, which is particularly problematic in clinical settings, where decisions must be transparent and justifiable. The inability to interpret model outputs can erode the trust between clinicians and patients, limiting adoption [114].

### Ethical and privacy concerns

Genomic data contain highly sensitive information that can reveal an individual’s identity, disease risk, and familial relationships. The risk of data breaches or unauthorized access can have far-reaching implications, including discrimination in the employment, insurance, and social contexts. Ensuring robust data protection is critical but challenging given the increasing sophistication of cyber threats [73].

The participants may not have fully understood the implications of sharing genomic information, particularly in the context of long-term storage or future use by third parties. These ethical concerns necessitate greater transparency and dynamic consent models that allow participants to update their preferences over time [115].

### Clinical integration limitations

The participants may not have fully understood the implications of sharing genomic information, particularly in the context of long-term storage or future use by third parties. These ethical concerns necessitate greater transparency and dynamic consent models that allow participants to update their preferences over time [116].

Although ML models can identify patterns and associations in genomic data, translating these findings into actionable clinical decisions is challenging. Many genomic variants identified using ML lack sufficient evidence to guide treatment, resulting in a gap between research and clinical practice. Bridging this gap requires interdisciplinary collaboration and validation studies to establish clinical utility [117].

### Regulatory and policy limitations

The absence of standardized guidelines for validating and approving ML models in genomics poses a major regulatory hurdle. Existing frameworks often fail to address the unique challenges of ML applications such as algorithmic transparency, reproducibility, and adaptation to evolving data. Regulatory frameworks vary widely across countries, which creates challenges for international collaboration. For example, data-sharing across borders is often constrained by different privacy laws and compliance requirements. This lack of harmonization impedes the global scalability of [118].

### Limitations in research and development

Although multi-omics approaches offer a comprehensive view of biological systems, their integration remains technically challenging. The lack of standardized formats, analytical pipelines, and computational tools hampers the effective combination of diverse data types such as genomics, transcriptomics, and proteomics. These limitations limit the ability to uncover complex disease mechanisms and identify possible therapeutic targets [119].

Genomic sequencing and downstream analyses remain expensive despite decreasing costs. This financial barrier limits access to precision medicine in resource-constrained settings and prevents healthcare inequities. Funding for genomic research and infrastructure development is critical for overcoming these barriers [120].

### Addressing challenges and moving forward

Genomic sequencing and downstream analyses remain expensive despite decreasing costs. This financial barrier limits access to precision medicine in resource-constrained settings and prevents healthcare inequities. Funding for genomic research and infrastructure development is critical for overcoming these barriers [121, 122].

Future advancements should focus on the development of interpretable ML models tailored to genomic applications. XAI techniques such as feature attribution and visualization tools can improve transparency and trust. Additionally, fairness-aware algorithms that mitigate biases in training data are essential for ensuring equitable outcomes [123].

Global regulatory bodies must collaborate to establish harmonized frameworks for genomic data protection and ML model validation. Dynamic consent models that allow participants to adjust their preferences over time should also be adopted to enhance ethical transparency [124].

Table 9 summarizes the key challenges, examples, solutions, and references in genomic data analysis, highlighting barriers and actionable strategies for advancing genomic research and precision medicine.

**Table 9:** Challenges, examples, solutions, and references in genomic data analysis (2022–2025)

**Category**	**Challenge**	**Examples**	**Solutions**	**Ref**
Data scarcity	Limited access to diverse genomic datasets; imbalance in variant representation	Rare disease datasets; bias towards European ancestry	Build globally diverse datasets; Use data augmentation and synthetic data generation techniques.	[125]
Data quality	Variability in sequencing platforms and errors in data	Inconsistent variant calling between platforms	Standardize sequencing protocols; Enhance preprocessing and noise-reduction algorithms.	[126]
Computational demands	Need for high-performance infrastructure for analyzing genomic data	High costs in low-resource settings	Develop optimized ML models; Leverage distributed and cloud computing.	[127]
Model transparency	Difficulty in interpreting “black-box” ML models	DL predictions lacking clinical rationale	Focus on XAI techniques; Incorporate visualization tools and feature attribution.	[128]
Ethical concerns	Privacy risks and challenges in informed consent	Data breaches; unclear secondary use policies	Implement dynamic consent models; Strengthen encryption and access control measures.	[129]
Clinical integration	Barriers in embedding genomic insights into healthcare workflows	Incompatibility with EHR systems; lack of clinician expertise	Upgrade EHR systems; Train healthcare professionals in genomic medicine.	[130]
Global disparities	Variability in data-sharing policies and standards across countries	Conflicting privacy regulations, limited collaboration	Harmonize global frameworks; Encourage international genomic research initiatives.	[131]
High costs	Financial barriers in genomic sequencing and research	Limited adoption in resource-constrained settings	Promote cost-efficient sequencing; Increase public and private funding for genomic research.	[132]

## Challenges and future directions

This section explores the key barriers and offers detailed insights into the directions for future research and applications.

### Key challenges

1. Data Scarcity and Quality Issues: Rare genetic disorders, by their very nature, affect a small fraction of the population, making it difficult to obtain large, high-quality datasets. ML models require substantial training data for robustness and accuracy [1].
  Moreover, the quality of genomic data is often compromised by noise, missing values, or inconsistencies in the sequencing methods. Low-quality data can introduce inaccuracies into the ML models, reducing their predictive power, and limiting their clinical applicability [60].
2. Ethical and Privacy Concerns: Ethical concerns arise around the collection, storage, and use of such data, particularly when participants may not fully understand how their information will be used [133].
  In addition, regulatory frameworks such as GDPR and HIPAA impose strict privacy requirements that conflict with the need for collaborative genomic research. Balancing the need for research and the protection of individual rights is an ongoing challenge, particularly as ML applications grow more sophisticated and demand larger datasets [81].
3. Computational Complexity and Scalability: Genomic data are inherently high dimensional, with millions of genetic variants per individual. This complexity increases exponentially when integrating multi-omics data or analyzing population-scale datasets. Training ML models on such data requires significant computational resources, including high-performance computing clusters and optimized algorithms. Small institutions or developing regions may lack access to these resources, creating disparities in their genomic research capabilities [134].
  Current algorithms often struggle to provide timely results without compromising accuracy. Furthermore, as datasets grow in size, the storage and processing requirements become increasingly challenging, highlighting the need for scalable solutions [135].
4. Model Interpretability and Trust: Many ML models, particularly DL architectures, are black-box models, which provide predictions without clear explanations. In clinical settings where trust and transparency are paramount, this lack of interpretability hinders adoption. Clinicians must understand why a model makes a particular prediction to ensure that the recommendation aligns with the medical knowledge and practices [136].

Table 10 presents a systematic comparison of the key challenges, methodologies, and outcomes across studies, underscoring the significant advancements in genomic research.

**Table 10:** Comparison of key challenges, methods, and results from different studies

**Key Applications**	**Key Challenges Addressed**	**Methods Used**	**Results**	**Ref**
Improved genomic data preprocessing	Data scarcity and noise in genomic datasets	Advanced preprocessing techniques for handling noise and missing values	Improved predictive accuracy in genomic analysis	[137]
Ethical and secure genomic data sharing	Ethical and privacy concerns in genomic research	GDPR-compliant data sharing frameworks and anonymization techniques	Enhanced data protection and ethical compliance	[133]
Efficient analysis of large genomic datasets	Computational complexity of high-dimensional genomic data	High-performance computing and optimized algorithms	Reduced computational time with high accuracy	[138]
Enhanced clinical decision-making using AI	Model interpretability in clinical applications	Development of interpretable ML models and XAI methods	Increased clinician trust and regulatory compliance	[139]
Multi-omics research in personalized medicine	Integration of multi-omics data	Multi-modal data harmonization using advanced integration techniques	Improved clinical applicability and multi-omics insights	[140]

### Future directions

1. Development of Universal and Diverse Genomic Dataset: Future studies should focus on creating large, diverse, and representative genomic datasets. International collaborations such as the Global Alliance for Genomics and Health (GA4GH) can help establish standardized protocols for data collection, storage, and sharing [141].
  Advancements in data augmentation techniques can mitigate data scarcity. Synthetic data generation using generative adversarial networks is a promising approach for creating realistic genomic datasets while preserving privacy [142].
2. Ethical and Secure Data Sharing Frameworks: Federated learning represents a key innovation in addressing privacy concerns while enabling collaborative research. In this approach, ML models are trained locally on decentralized data, with only the model updates shared among institutions [42].
  Blockchain technology offers another solution by providing immutable access to data and usage records. By creating transparent and auditable trails, blockchain can enhance trust among stakeholders, while preventing unauthorized data modifications [94].
3. Advancements in XAI and Trustworthy Models: XAI is critical for bridging the gap between ML models and clinical practice. Future research should focus on developing XAI techniques that are specifically tailored for genomic applications. Feature attribution methods, which highlight genetic variants contributing to a prediction, can help clinicians understand the rationale behind model outputs [143].
4. Integration of Multi-Omics and Real-Time Analysis: The integration of multi-omics data, including genomics, transcriptomics, and proteomics, will be the cornerstone of future genomic research. Hybrid ML models capable of analyzing diverse data type will provide a more comprehensive understanding of disease mechanisms [98].
5. Global Collaboration and Interdisciplinary Research: Addressing the challenges of ML for GBPM requires collaboration across disciplines, including bioinformatics, ML, clinical medicine, and policy. Interdisciplinary teams can bridge the gap between technological innovation and real-world applications [144].
6. Regulatory and Governance Frameworks: Global regulatory bodies must collaborate to establish standardized guidelines for ML applications in genomics. These frameworks should address issues such as transparency, reproducibility, and fairness while balancing innovation with ethical considerations [145].

**Figure 8:**
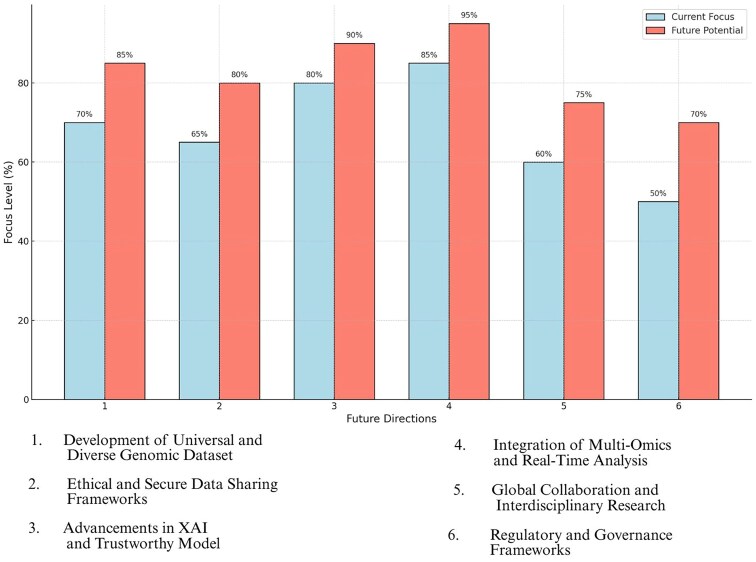
Comparison of current focus and future potential.

Figure 8 compares the current focus and future potential across six key directions in genomic research and ML [14, 146–149].

## Recommendations

This section outlines the key recommendations for addressing current challenges and advancing the field.

### Enhancing data diversity and quality

One of the most pressing requirements of GBPM is the development of large, diverse, and globally representative genomic datasets. Current datasets are heavily biased toward populations of European ancestry, limiting the generalizability of the ML models to other ethnic groups. Future initiatives should prioritize the inclusion of underrepresented populations to ensure equitable healthcare outcomes [150].

### Advancing ML methodologies

A major limitation arises from the tendency of XAI frameworks to oversimplify feature attributions in highly non-linear models such as deep neural networks and ensemble classifiers. These models can capture complex, non-additive interactions among features, but explanation methods like SHAP or LIME may attribute importance in a linear or additive fashion, which can lead to misleading or biologically implausible interpretations. For instance, a gene may appear important in isolation according to an XAI metric but may only have functional relevance in the context of a pathway or regulatory module. Moreover, explanations are often sensitive to model perturbations and lack consistency across different training instances, raising concerns about their robustness and reproducibility in high-stakes domains like precision medicine. Addressing these limitations will require the development of genomics-aware XAI methods that can model complex interactions and reflect underlying biological mechanisms more faithfully [13, 107, 151].

### Strengthening ethical and privacy frameworks

Obtaining informed consent is a dynamic process that must evolve with the expanding applications of genomic data. Future frameworks should adopt dynamic consent models allowing participants to update their preferences when new research applications emerge [136].

Obtaining informed consent is a dynamic process that must evolve with the expanding applications of genomic data. Future frameworks should adopt dynamic consent models allowing participants to update their preferences when new research applications emerge [95].

### Improving computational infrastructure and scalability

The growing scale of genomic data necessitates advancements in high-performance computing. Distributed computing frameworks, cloud-based platforms, and optimized algorithms are critical for managing large-scale datasets and performing real-time genomic analyses. Investments in computational infrastructure will ensure that institutions worldwide participate in precision medicine research.

Automated data pre-processing, variant annotation, and ML pipeline execution can significantly reduce the time required for genomic analysis. Future pipelines should incorporate real-time processing capabilities to enable applications such as rapid neonatal diagnostics or time-sensitive cancer therapies [152].

### Integration with emerging technologies

The integration of ML with genome-editing technologies like CRISPR- Cas9 has immense potential for therapeutic innovation. ML models can optimize guide RNA design, predict off-target effects, and identify novel gene editing targets. Future research should explore these synergies to accelerate the development of precision therapeutics [153]. Single-cell sequencing technology provides unparalleled insights into cellular heterogeneity. ML models should be developed to analyze single-cell datasets to enable the identification of rare cell populations and their roles in disease progression. These advancements can revolutionize personalized medicine, particularly in oncology and immunotherapy [154]. One notable tool is scVI (single-cell Variational Inference), which uses a deep generative model based on variational autoencoders to model the gene expression distribution across cells. It provides a robust latent space representation while correcting for batch effects and capturing biological variation [155]. Another emerging technique is scGNN, which introduces a graph neural network-based approach for learning gene-cell relationships, thereby enhancing clustering accuracy and data imputation [156]

**Figure 9:**
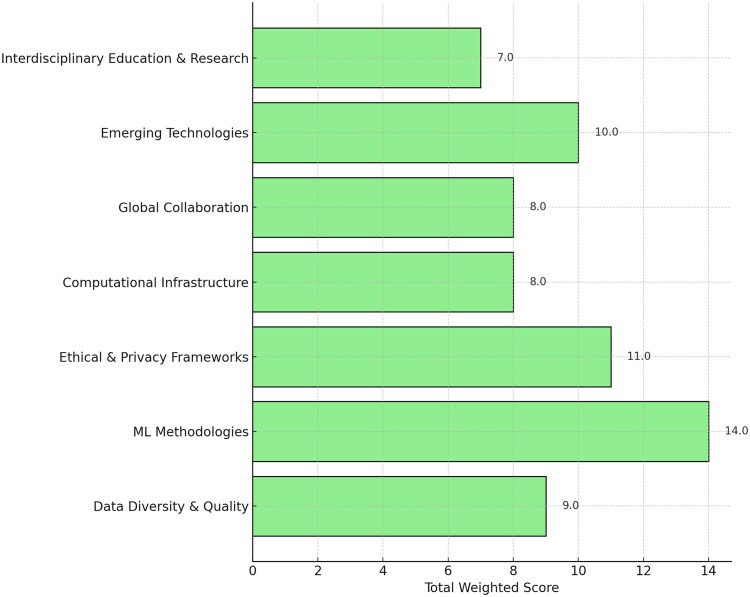
Emphasizing total contributions across categories.

Figure 9 highlights the weighted contributions across key areas in GBPM, emphasizing data diversity, advanced ML methodologies, ethical frameworks, computational tools, global collaboration, and interdisciplinary education [157–160].

## Discussion and interpretation of results

This section synthesizes the key takeaways from this review, highlighting the progress made, obstacles encountered, and future directions required to advance this field.

### Summary of key advancements

The advent of NGS and multi-omics integration has been pivotal in enabling GBPM. These technologies have provided unprecedented resolution for understanding the genetic and molecular basis of diseases. Clinical applications, such as rapid whole-genome sequencing for neonatal disorders and personalized cancer therapies, exemplify the transformative impact of these advancements.

ML has emerged as a critical enabler in precision medicine, enhancing our ability to analyze high-dimensional genomic data, identify pathogenic variants, and predict disease phenotypes. The integration of ML with explainable XAI techniques has improved the interpretability of predictions, fostering trust and adoption in clinical settings. ML-driven innovations in drug repurposing and therapeutic target identification have accelerated the development of personalized treatments.

### The path forward

The future of GBPM lies in its ability to deliver equitable healthcare outcomes. This requires concerted efforts to build global data-sharing frameworks, harmonize regulatory standards, and ensure access to genomic technologies in low- and middle-income countries. Collaborative initiatives such as international genomic consortia and public-private partnerships play a pivotal role in achieving these goals. Integrating emerging technologies, such as CRISPR-based genome editing, single-cell sequencing, and federated learning, with ML holds immense promise for advancing precision medicine.

Future advancements in ML methodologies, including fairness-aware algorithms, multi-modal data integration, and XAI will enhance the accuracy, interpretability, and reliability of genomic analyses. These innovations will ensure that ML models are scientifically robust and ethically and socially responsible.

## Conclusion

By facilitating more precise diagnosis, customized therapies, and quicker drug development, ML is propelling revolutionary advancements in GBPM for rare genetic diseases. ML has greatly enhanced disease classification and biomarker identification through the development of models that can analyze complicated and high-dimensional genomic data. However, real-world implementation remains constrained by issues such as unbalanced and small datasets, computing complexity, ethical dilemmas, and model comprehensibility. To overcome these obstacles and develop trust in healthcare environments, explainable AI must be integrated into strong data-sharing frameworks and fairness-aware algorithms. This review emphasizes the necessity of cross-disciplinary collaboration to address these issues, and presents a thorough research roadmap that outlines practical strategies for transforming ML developments into impactful and equitable precision healthcare.

**Key Points**

- Present a comprehensive and up-to-date assessment of ML’s current status in GBPM.
- Seeks to address literature gaps, focusing on real-world challenges and ethical considerations.
- Identify knowledge and practice gaps preventing ML from reaching its full potential in this area.
- The review serves as a roadmap for future research by proposing actionable recommendations.
- Presents a comprehensive and up-to-date assessment of machine learning’s ML’s current status in genome-based precision medicine
- Seeks to address literature gaps, focusing on real-world challenges and ethical considerations
- Identifies knowledge and practice gaps preventing ML from reaching its full potential in this area
- Serves as a roadmap for future research by proposing actionable recommendations.
